# Practice Patterns and Outcomes of Patients With Retained Gastric Food Content Encountered During Endoscopy

**DOI:** 10.14309/ctg.0000000000000559

**Published:** 2023-01-24

**Authors:** Emily Lin, Yinglin Gao, Andrew Wright, John J. Kim

**Affiliations:** 1 Division of Gastroenterology, Loma Linda University Health, Loma Linda, California, USA.

**Keywords:** gastric, retained, food, gastroparesis, aspiration, endoscopy

## Abstract

Retained gastric food content encountered during upper endoscopy may reduce diagnostic accuracy and increase the risk of aspiration. The aim of this study was to evaluate endoscopists' practice patterns and clinical outcomes in patients with retained gastric food content encountered during endoscopy.

**METHODS:**

Consecutive patients with retained gastric food content during first-time endoscopy at Loma Linda University Health (January 2016–March 2021) were identified. Primary endpoints were a complete examination (deep duodenal intubation) and 30-day postprocedural respiratory adverse events.

**RESULTS:**

Of 17,868 patients undergoing endoscopy, 629 (3.5%) (mean age 55 ± 17 years) met inclusion criteria. Moderate sedation was performed in 506 (80.4%), anesthesiologist-assisted sedation in 16 (2.5%), and general anesthesia in 107 (17.0%) patients. 534 (84.9%) patients received a complete examination, and endoscopist-specific completion rates varied by quintile among 26 endoscopists (range 70.3%–98.0%, *P* < 0.0001). Large food gastric content decreased (adjusted odds ratio [aOR] 0.3, 95% confidence interval [CI] 0.2–0.4) while obtaining mucosal biopsies increased (aOR 2.5, 95% CI 1.4–4.7) the likelihood of complete examination after adjusting for endoscopist-specific completion rates. Subsequently, 58 (9.2%) patients required repeat endoscopy within 30 days. During follow-up, 41 (6.5%) patients developed respiratory adverse events including 21 (3.3%) requiring ventilatory support. Hospitalized patients (aOR 37.8, 95% CI 4.9–289.0) compared with outpatients and large compared with small gastric food content (aOR 2.1, 95% CI 1.1–4.2) increased the likelihood of respiratory adverse events.

**DISCUSSION:**

Although deep duodenal intubation was achieved in most patients receiving endoscopy, the rate of complete examination varied among individual endoscopists and the extent of food burden. Respiratory adverse events occurred almost exclusively in hospitalized patients and were associated with high morbidity including half developing respiratory failure.

## INTRODUCTION

Retained gastric food contents are frequently encountered during upper endoscopy. Depending on the patient population and clinical setting, retained gastric contents have been reported in 0.3%–19.0% of upper endoscopies (1–3). Given the rising incidence and morbidity of gastroparesis, the incidence of retained gastric content encountered during upper endoscopy may also increase (4). Retained gastric food content may lead to suboptimal examination, affecting accurate diagnosis and delaying therapeutic interventions. This in turn may lead to a need for repeat procedures, which is associated with additional procedural risk and cost. Finally, aspiration of gastric food content during the period of sedation may lead to potentially life-threatening respiratory adverse events including aspiration pneumonia and chemical pneumonitis (5–7).

While previous studies have primarily focused on identifying risk factors for retained gastric food content (1), the effect of retained gastric contents during endoscopy on diagnostic accuracy, outcomes of therapeutic interventions, and patient safety remains unknown. We hypothesize that retained gastric content encountered during endoscopy may reduce diagnostic accuracy and increase the risk of adverse respiratory events related to aspiration. Furthermore, given the lack of specific recommendations from society guidelines, substantial variability among individual endoscopist practice patterns is expected. The primary aims of this study were to evaluate endoscopists' practice patterns by assessing the proportion of patients who were able to undergo a complete endoscopic examination to the second portion of the duodenum despite retained food contents and the incidence of respiratory adverse events after endoscopy. Predictors associated with complete examination and respiratory adverse events were also evaluated in this patient population.

## METHODS

### Study design

Loma Linda University Institutional Review Board approval was obtained before initiating this retrospective study. Consecutive patients older than 18 years with retained gastric food content observed during first-time upper endoscopy at Loma Linda University Medical Center from January 2016 to March 2021 were included in the study.

Upper endoscopy was performed after >6 hours of fasting of solids and >2 hours from clear liquid based on institutional gastrointestinal (GI) laboratory and anesthesia protocol. Search terms (“food,” “retention,” “retained,” “bezoar,” and “debris”) were used to identify the patient population meeting study criteria from an internal endoscopy database. Procedure reports were subsequently reviewed by 2 independent researchers to verify the presence of retained gastric contents encountered during endoscopy. Exclusion criteria included patients who were pregnant, had received prior foregut surgery, or were already on mechanical ventilation before planned endoscopy. In patients who had multiple procedures, the first procedure at the institution during the study period was considered the index procedure. Medical records including radiologic studies and endoscopic reports were carefully reviewed to characterize the patient's clinical course after endoscopy. Data including demographic information, endoscopist performing the procedure, American Society of Anesthesiologists classification, inpatient vs outpatient status, location of procedure, indication for procedure, type of sedation or anesthesia, and therapeutic interventions performed were collected. Large gastric food burden was defined as large amounts of gastric contents unable to be cleared after endoscopic lavage, which was confirmed by an independent review of endoscopic photographs. Small gastric food burden was defined as gastric content mostly cleared after endoscopic lavage. Retained esophageal food content was defined as the presence of concomitant esophageal food content in addition to gastric contents. Blood content alone in patients with upper GI bleeding was not considered retained gastric food content.

### Study endpoints

Primary endpoints included complete examination defined as successful endoscopic advancement to the second portion of the duodenum and respiratory adverse events within 30 days after endoscopy. Respiratory adverse events included any episode of acute respiratory symptoms associated with abnormal radiographic findings after endoscopy. Aspiration pneumonia was defined as the development of respiratory adverse event necessitating antimicrobial therapy. Secondary outcomes included incidence of repeat endoscopy, 30-day hospitalization among those who received outpatient endoscopy, and 30-day mortality after index endoscopy. Repeat endoscopy was defined by the need for repeat procedure within 30 days of the index procedure because of suboptimal examination or inability to perform intended therapy.

### Statistical analysis

Continuous variables were reported as mean ± SD, and categorical variables were reported as frequency (%). Individual endoscopist's complete examination rates were calculated and then categorized into quintiles in relation to other endoscopists. Univariate logistic regression analyses were used to determine associations between baseline demographic factors and primary outcomes. Significant covariates with *P* value <0.1 in univariate analysis were included in multivariate logistic regression analyses to derive adjusted odds ratios (aORs) and confidence intervals (CIs). All significance tests were 2-sided, and the significance level was set at 0.05. Statistical analyses were performed using SAS software, version 9.4 (SAS Institute, Cary, NC).

## RESULTS

### Study population

**Figure 1.**
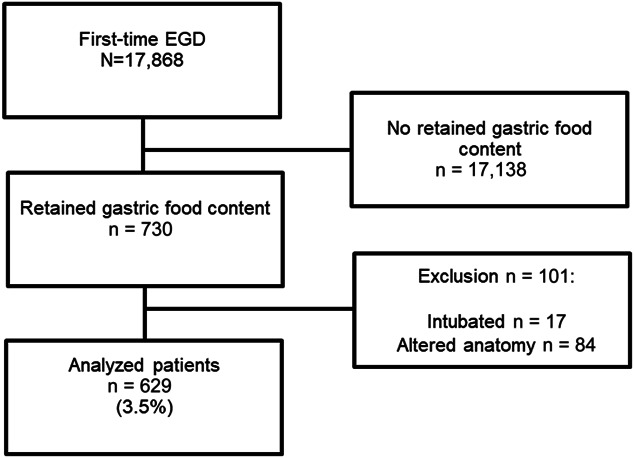
Flowchart of patient selection. EGD, esophagogastroduodenoscopy.

During the study period, 730 (4.1%) of 17,868 patients who received first-time upper endoscopy at Loma Linda University Medical Center were found to have retained gastric food content. After excluding patients who had altered surgical anatomy or who were already mechanically ventilated before the procedure, 629 (3.5%) met inclusion criteria (Figure 1). The mean age of patients was 55 ± 17 years, 310 (49.2%) were male, and 283 (44.9%) received endoscopy during hospitalization. Furthermore, 30 (4.8%) patients had a previous diagnosis of gastroparesis before outpatient (21, 3.3%) or inpatient (9, 1.4%) endoscopy; 9 received prokinetics before the procedure. A median of 20 (range 1–77) procedures demonstrating retained gastric food content were performed by 26 endoscopists. The most common primary indications for endoscopy included dysphagia in 143 (22.7%), upper GI bleeding in 133 (21.1%), and vomiting in 75 (12.0%) (Table 1). Procedural characteristics are summarized in Table 2. Moderate sedation, anesthesiologist-assisted sedation, and general anesthesia were performed in 506 (80.5%), 16 (2.5%), and 107 (17.0%) patients, respectively. The median duration of procedure was 22 minutes (range 2–180). Large and small gastric food burden was observed in 256 (40.7%) and 373 (59.3%) patients, respectively. Seventy-five (11.9%) patients were found to have some degree of retained food in the esophagus including 10 with achalasia. One hundred sixty-three (25.9%) patients received planned therapeutic interventions including esophageal dilation in 79 (12.6%), nonvariceal hemostasis in 22 (3.5%), and variceal band ligation in 13 (2.1%). Furthermore, 238 (37.8%) patients received mucosal biopsies including biopsies of the esophagus in 88 (14.0%), stomach in 175 (27.8%), and duodenum in 91 (14.5%) (Table 2).

**Table 1.** Baseline characteristics of patients with gastric food content encountered during endoscopy

	N = 629
Age, yr, mean ± SD	54.8 ± 16.5
Male, n (%)	310 (49.2)
Race/ethnicity, n (%)	
White	469 (74.6)
African American	65 (10.3)
Latino	43 (6.8)
Asian	33 (5.2)
Others	19 (3.0)
ASA class, n (%)	
1	48 (7.6)
2	270 (42.9)
3	288 (45.8)
4	23 (3.7)
History of gastroparesis, n (%)	30 (4.8)
Primary indications for EGD, n (%)	
Dysphagia	143 (22.7)
Upper GI bleeding	133 (21.1)
Vomiting	75 (12.0)
Dyspepsia	58 (9.2)
Screen for varices	64 (10.2)
GERD symptoms	42 (6.7)
Anemia	29 (4.6)
Diarrhea	16 (2.5)
History of gastric ulcer	15 (2.4)
Weight loss	11 (1.7)
Others	43 (6.8)
Hospitalization, n (%)	283 (44.9)

ASA, American Society of Anesthesiologist; EGD, esophagogastroduodenoscopy; GERD, gastroesophageal reflux disease; GI, gastrointestinal.

**Table 2.** Procedure characteristics

	N = 629
Procedure location, n (%)	
Endoscopy laboratory	570 (90.6)
Emergency department	6 (1.0)
Intensive care unit	49 (7.8)
Operating room	4 (0.6)
Anesthesia, n (%)	
Moderate sedation	506 (80.4)
Anesthesiologist-assisted sedation	16 (2.5)
General anesthesia	107 (17.0)
Mucosal biopsies, n (%)	238 (37.8)
Esophagus	88 (14.0)
Stomach	175 (27.8)
Duodenum	91 (14.5)
Esophageal food content, n (%)	75 (11.9)
Endoscopic interventions, n (%)	163 (25.9)
Esophageal dilation	79 (12.6)
Nonvariceal hemostasis	22 (3.5)
Stent placement	14 (2.2)
Variceal band ligation	13 (2.1)
Endoscopic resection	12 (1.9)
Submucosal injection	11 (1.7)
Percutaneous endoscopic gastrostomy	10 (1.6)
Others	3 (0.0)
Estimate of gastric food content, n (%)	
Large	256 (40.7)
Small	373 (59.3)
Median length of procedure (range), min	22 (2–180)

### Clinical outcomes

Of the 629 patients with retained gastric contents encountered during endoscopy, 534 (84.9%) received complete examination to the second portion of duodenum (Table 3). Fifty-eight (9.2%) patients required repeat endoscopy within 30 days of index procedure because of suboptimal examination or postponed intervention. Forty-two (6.7%) patients developed adverse events after endoscopy including 41 (6.5%) who received inpatient and 1 (0.2%) who received outpatient procedures. Of the 42 patients, 41 (6.5%) with retained gastric contents experienced a respiratory adverse event within 30 days, while 1 (0.2%) patient developed an esophageal microperforation after esophageal stricture dilation and was treated conservatively with antibiotics. Respiratory adverse events included aspiration pneumonia requiring antibiotics in 30 (4.8%) patients, respiratory events not requiring antibiotics in 10 (1.6%) patients, and airway obstruction due to obstructive mucous plugging in 1 (0.2%) patient. Of the 41 patients who developed respiratory adverse events, 21 (51.2%) developed respiratory failure requiring intensive care for ventilatory support and 10 (23.8%) died during the hospitalization course. Only 1 patient who received outpatient procedure developed respiratory adverse event. In that case, a 75-year-old woman with nonalcoholic steatohepatitis cirrhosis who was undergoing screening endoscopy for esophageal varices was found to have large amounts of food contents in her stomach. She was hospitalized 2 days later for tense ascites and dyspnea with imaging consistent with chemical pneumonitis and later recovered with diuretics and oxygen therapy.

**Table 3.** Clinical outcomes

	N = 629
Complete examination to the second portion of duodenum, n (%)	534 (84.9)
Repeat EGD, n (%)	58 (9.2)
Mean days to repeat EGD ± SD	8.8 ± 8.3
Total adverse events related to endoscopy, n (%)	42 (6.7)
Respiratory adverse events	41 (6.5)
Aspiration pneumonia	30 (4.8)
Airway obstruction	1 (0.2)
Esophageal perforation	1 (0.2)
30-d hospitalization, n (%)^a^	18/346 (5.2)
30-d mortality, n (%)	15 (2.4)

EGD, esophagogastroduodenoscopy.

a Rate of 30-day hospitalization seen in outpatient cases.

Of the 346 patients who received outpatient endoscopy, 18 (5.2%) required hospitalization within 30 days including 1 for respiratory adverse events. Overall, 15 (2.4%) patients died within 30 days of the procedure.

### Predictors of complete examination

The median rate of complete examination among 26 endoscopists was 85.1% (interquartile range, 77%–97%). On univariate analysis, the degree of gastric food contents (74.6% vs 92.2%, *P* < 0.0001), duration of procedure ≥20 minutes (87.0% vs 82.0%, *P* = 0.09), obtaining mucosal biopsies (93.7% vs 79.5%, *P* < 0.0001), and complete examination rate per endoscopists by quintile (70.3%–98.0%, *P* < 0.0001) were associated with complete examination to the second portion of the duodenum (Table 4). On multivariate analysis, large compared with small gastric food burden was associated with a decreased likelihood of complete examination (aOR 0.26, 95% CI 0.16–0.43), while mucosal biopsies obtained during endoscopy was associated with an increased likelihood of complete examination (aOR 2.54, 95% CI 1.38–4.69). Furthermore, when analyzing individual endoscopist complete examination rates by quintiles, patients who received procedures by endoscopists in the first (aOR 15.64, 95% CI 4.58–53.39), second (aOR 3.81, 95% CI 1.64–8.87), and third quintiles (aOR 2.29, 95% CI 1.18–4.47) were more likely to receive a complete examination compared with those who received procedures in the fifth quintile.

**Table 4.** Predictors of deep duodenal intubation in patients with gastric food content encountered during endoscopy

	Univariate		Multivariate^a^	
	N = 629 n/N (%)	*P* value	aOR (95% CI)	*P* value
Age, yr				
<40	112/133 (84.2)	0.78		
40–60	199/237 (84.0)			
>60	223/259 (86.1)			
Sex				
Male	256/310 (82.6)	0.11		
Female	278/319 (87.1)			
ASA				
1	40/48 (83.3)	0.92		
2	227/270 (84.1)			
3	247/288 (85.8)			
4	20/23 (87.0)			
Hospitalization				
Inpatient	244/283 (86.2)	0.42		
Outpatient	290/346 (83.8)			
Anesthesia				
Moderate sedation	426/506 (84.2)	0.44		
Anesthesiologist-assisted sedation	13/16 (81.3)			
General anesthesia	95/107 (88.8)			
Upper GI bleeding				
Yes	118/133 (88.6)	0.18		
No	416/496 (83.9)			
Esophageal food content				
Yes	61/75 (81.3)	0.36		
No	473/554 (85.4)			
Gastric food content				
Large	191/256 (74.6)	<0.0001	0.26 (0.16–0.43)	<0.0001^c^
Small	343/373 (92.0)			
Mucosal biopsies				
Biopsies	223/238 (93.7)	<0.0001	2.54 (1.38–4.69)	0.003^c^
No biopsies	311/391 (79.5)			
Interventions other than biopsies				
Interventions	134/163 (82.2)	0.27		
No interventions	400/466 (85.2)			
Duration of procedure				
≥20 min	320/368 (87.0)	0.09	0.73 (0.45–1.18)	0.19
<20 min	214/261 (82.0)			
Deep duodenal intubation rate by endoscopist^b^				
First quintile	127/130 (98.0)	<0.0001	15.64 (4.58–53.39)	<0.0001^c^
Second quintile	106/114 (93.0)		3.81 (1.64–8.87)	0.002^c^
Third quintile	113/130 (87.0)		2.29 (1.18–4.47)	0.01^c^
Fourth quintile	98/127 (77.0)		1.08 (0.59–1.98)	0.83
Fifth quintile	90/128 (70.3)		Reference	

aOR, adjusted odds ratio; ASA, American Society of Anesthesiologists; CI, confidence interval; GI, gastrointestinal.

a Multivariate logistic regression analysis was conducted when significant covariates were seen in univariate model.

b Rate of examination completeness was stratified into quintiles based on how often endoscopists achieved deep duodenum intubation.

c Signifies statistical significance.

### Predictors of respiratory adverse events

On univariate analysis, the American Society of Anesthesiologists class (range 0%–17.4%, *P* = 0.002), inpatient status (14.1% vs 0.3%, *P* < 0.0001), upper GI bleeding (16.7% vs 3.8%, *P* < 0.0001), degree of gastric food content (10.2% vs 4.0%, *P* = 0.002), and obtaining mucosal biopsies during procedure (3.8% vs 8.2%, *P* = 0.03) were associated with increased respiratory adverse events (Table 5). Conversely, the type of anesthesia, endoscopic interventions other than biopsies, concurrent esophageal food content, and the duration of the procedure were not associated with odds of respiratory adverse events. On multivariate analysis, inpatient status during endoscopy (aOR 37.78, 95% CI 4.94–288.99) and large gastric food content (aOR 2.14, 95% CI 1.09–4.20) were associated with an increased likelihood of respiratory adverse events.

**Table 5.** Pulmonary adverse events in patients with gastric food content encountered during endoscopy

	Univariate		Multivariate^a^	
	N = 629 n/N (%)	*P* value	aOR (95% CI)	*P* value
Age, yr				
<40	10/133 (7.5)	0.79		
40–60	16/237 (6.8)			
>60	15/259 (5.8)			
Sex				
Male	23/310 (7.4)	0.37		
Female	18/319 (5.6)			
ASA				
1	0/48 (0.0)	0.002	<0.001 (<0.001–999.99)	0.97
2	10/270 (3.7)		0.88 (0.23–3.34)	0.85
3	27/288 (9.4)		0.76 (0.23–2.47)	0.64
4	4/23 (17.4)		Reference	
Hospitalization				
Inpatient	40/283 (14.1)	<0.0001	37.78 (4.94–288.99)	0.0005^b^
Outpatient	1/346 (0.3)			
Anesthesia				
Moderate sedation	35/506 (6.9)	0.50		
Anesthesiologist-assisted sedation	0/16 (0.0)			
General anesthesia	6/107 (5.6)			
Upper GI bleeding				
Yes	22/133 (16.6)	<0.0001	1.66 (0.81–3.37)	0.17
No	19/496 (3.8)			
Gastric food content				
Large	26/256 (10.2)	0.002	2.14 (1.09–4.20)	0.02^b^
Small	15/373 (4.0)		Reference	
Esophageal food content				
Yes	6/75 (8.0)	0.54		
No	35/554 (6.3)			
Mucosal biopsies				
Biopsies	9/238 (3.8)	0.03	0.60 (0.27–1.36)	0.22
No biopsies	32/391 (8.2)			
Interventions other than biopsies				
Interventions	8/163 (4.9)	0.33		
No interventions	33/466 (7.1)			
Deep duodenal intubation				
Yes	32/534 (6.0)	0.21		
No	9/95 (9.5)			
Duration of procedure				
≥20 min	25/368 (6.8)	0.74		
<20 min	16/261 (6.1)			

aOR, adjusted odds ratio; ASA, American Society of Anesthesiologists; CI, confidence interval; GI, gastrointestinal.

a Multivariate logistic regression analysis was conducted when significant covariates were seen in univariate model.

b Signifies statistical significance.

## DISCUSSION

In this single-center US study of patients undergoing upper endoscopy, unexpected gastric food content occurred in approximately 1 in 30 procedures after excluding those with anatomic foregut alterations or prior airway protection. Although deep duodenal intubation was achieved in most (84.8%) patients, substantial variation in complete examination rates was observed among individual endoscopists. The presence of large food burden was associated with decreased odds while obtaining mucosal biopsies during procedure was associated with increased odds of complete examination after adjusting for endoscopist-specific completion rate. Finally, respiratory adverse events occurred in 7% of the study population but occurred almost exclusively in hospitalized patients receiving endoscopy and were more likely to occur with large compared with small gastric food content.

Society guidelines recommend fasting for a minimum of 6 hours from solids before endoscopy with sedation to allow sufficient time for gastric emptying (3,8,9). Despite adhering to guidelines, patients receiving endoscopy frequently have unexpected retained gastric food contents that may compromise diagnostic and therapeutic goals. Furthermore, retained solid gastric food content raises concern for poor clinical outcomes including procedure-related respiratory adverse events and aborted or repeat endoscopic procedures. Prior studies have focused largely on identifying factors associated with gastric food content during endoscopy including opioid use, postsurgical anatomy, and medical comorbidities such as diabetes, gastroparesis, and cirrhosis (1–3,6,7,10,11). However, clinical outcomes including the risk of procedure-related adverse events are not well-described. Furthermore, there is a lack of studies investigating how retained gastric contents directly affect endoscopist practice patterns including endoscopists' ability to perform complete endoscopic examinations and therapeutic interventions. Given the lack of society guidelines providing a clear recommendation on how to proceed in such situations, a large variation in endoscopist practice patterns is expected.

In our study, 534 (84.8%) patients received complete examination despite most of them (82.9%) receiving endoscopy without preprocedural airway intubation. A large portion of patients received therapeutic interventions (25.9%) or mucosal biopsies (37.8%) despite suboptimal conditions. Although most patients received complete examination to the distal duodenum, substantial variation was observed among individual endoscopists. Notably, when endoscopists were stratified into quintiles based on examination completion rates, patients who underwent procedures performed by endoscopists in the highest quintile had a 16-fold increased likelihood of complete examination compared with those in the lowest quintile. Furthermore, large compared with small gastric food burden was associated with a greater than 70% reduction in likelihood of complete examination, which aligns with endoscopists' perception that large gastric burden elevates the risk of aspiration. Large amounts of gastric food content may also preclude detailed mucosal visualization, leading to missed lesions including neoplasms (3). As such, 58 (9.2%) patients received repeat endoscopy at a mean of 8.8 ± 8.3 days after index endoscopy because of suboptimal initial examination. Notably, even in the subgroup of patients with small gastric food content that were mostly cleared during endoscopy, non-negligible portions failed to receive a complete examination to the second portion of the duodenum (8.0%). Finally, our results also revealed that obtaining mucosal biopsies during the procedure despite the presence of retained gastric food was associated with a nearly 3-fold increased likelihood of complete examination, likely representing the endoscopist's overall comfort level and intention to complete the examination.

Of interest, the type of anesthesia used during endoscopy demonstrated the lack of association with complete examination. One might expect that the endoscopist would be more inclined to proceed with a complete examination when airway protection is secured with general anesthesia or anesthesiologist-assisted procedures. The lack of association between sedation type and complete examination in our study may be explained by the relatively low proportions of anesthesiologist-assisted cases compared with moderate sedation cases. Finally, the presence of concomitant food contents in the esophagus did not seem to significantly affect the endoscopist's decision to proceed with complete examination. Potential factors influencing the endoscopists' decision to proceed were likely based on individual-level benefit-risk assessments (i.e., individual patient's risk of aspiration, urgency of endoscopic examination) but was ultimately beyond the scope of this study.

After endoscopy, 41 (6.5%) patients experienced respiratory adverse events including 30 (4.8%) with aspiration pneumonia, necessitating antimicrobial therapy. Notably, 21 (3.3%) patients required intensive care for ventilatory support because of procedure-related respiratory adverse event. Overall, the incidence of respiratory events in our study was substantially higher than that noted in prior studies involving patients undergoing endoscopy. In a prospective observational study conducted in Germany that included 6,516 patients who received outpatient endoscopy with endoscopist-directed sedation with or without colonoscopy, 17 (0.3%) patients developed signs of respiratory symptoms suspicious for infection during a 24-hour phone follow-up, though none required hospitalization (12). In a single-center Italian study that included 3,001 inpatient and outpatient upper endoscopy cases performed under monitored anesthesia care, immediate intraprocedural desaturation, respiratory depression, and aspiration were documented in 46 (1.5%), 5 (0.2%), and 6 (0.2%) patients, respectively (13). Other studies have demonstrated that aspiration is rare (<0.2%) during endoscopic procedures performed under general anesthesia (7). Although it is possible that the definition of respiratory adverse event used in this study may have captured pulmonary disease unrelated to the procedure, the high incidence of those who developed respiratory failure is nevertheless surprising, considering the infrequent incidence of respiratory adverse events historically observed in the general population undergoing endoscopy.

Respiratory adverse events were observed almost exclusively in patients who received endoscopy during hospitalization. Specifically, hospitalized patients who received endoscopy demonstrated a 39-fold increased risk of developing a respiratory adverse event compared with those who received endoscopy in outpatient settings. Although active underlying illness or medical comorbidities in hospitalized patients may be a confounder of pulmonary disease, the magnitude of risk for respiratory event was strikingly high. Only 1 (0.3%) patient with significant comorbidity (i.e., decompensated cirrhosis) developed a respiratory adverse event after receiving outpatient endoscopy, suggesting that proceeding with endoscopic examination in the outpatient setting despite the presence of gastric food contents may be safe. In addition, the risk of respiratory adverse event more than doubled with large compared with small gastric food contents (aOR 2.14, 95% CI 1.09–4.20) as expected. Conversely, receiving endoscopic intervention other than biopsies and type of anesthesia used for endoscopy were not associated with odds of respiratory adverse events. Of interest, our results revealed that patients with retained gastric contents receiving general anesthesia did not have reduced odds of pulmonary adverse events compared with those receiving anesthesia-assisted or moderate sedation. Although unclear, the risk of pulmonary adverse events may persist despite elective intubation in this population, similar to patients with massive GI bleeding with frequent retained gastric blood content who receive prophylactic intubation before endoscopy (14). Finally, upper GI bleeding, which is typically an indication for urgent or emergent endoscopy (5,15,16), was not associated with an increased risk of pulmonary events.

The results of this study have clinical implications. The high incidence and morbidity related to respiratory adverse events after endoscopy in hospitalized patients with retained gastric food content are alarming. Interventions to reduce the risk of pulmonary adverse events in this patient population are needed. First, before planned endoscopy, a careful risk assessment for predictors of retained gastric food contents such as symptoms of gastroparesis, a history of cirrhosis, or opioid use should be performed. A longer duration of fasting than the current recommendation of 6 hours before the planned endoscopy in patients with suspected abnormal gastric emptying should be implemented as suggested by society guidelines (17). This would be particularly important in hospitalized patients receiving endoscopy, given the high incidence of pulmonary adverse event with retained gastric food content. Furthermore, liberal use of preprocedural prokinetic medications or nasogastric lavage—techniques typically reserved for patients undergoing endoscopy for upper GI bleeding—should be considered to reduce the risk of retained food content (8). Emerging studies have also demonstrated the application of bedside point-of-care ultrasound to assess for retained gastric food content before administration of sedation for minor procedures (18). By identifying predictors for endoscopy-related adverse events, our findings may also help guide the endoscopist's intraprocedural decision-making. Once gastric food content is encountered during endoscopy, the decision to complete or abort the remainder of the procedure can be made on an individual basis based on the risk of developing respiratory adverse events. Other intraprocedural interventions such as elevating the head of the bed, avoiding deep sedation, and using minimal air insufflation to reduce the risk of aspiration may be useful, but were not formally evaluated in this study.

To our knowledge, this is the first study to evaluate the effect of retained gastric contents on clinical outcomes and endoscopist practice patterns in patients specifically undergoing upper endoscopy. A strength of this study is the generalizability of our findings, which included evaluation of patients undergoing upper endoscopy in both the inpatient and outpatient settings under mostly endoscopist-directed moderate sedation. Furthermore, endoscopic practice pattern of 26 endoscopists with variable training background and experience spanning a study period of 15 years were examined. Limitations of the study include its retrospective study design. Accurate data on adherence and duration of fasting before endoscopy was not consistently available for analysis. Furthermore, categorization on the degree of food retention was determined by qualitative assessments made by individual endoscopists, which can lead to subjectivity. To minimize any potential inconsistency, procedural documentation assessing the quantity of retained food and photodocumentations of every procedure were independently reviewed. In addition, obtaining data on percentage of gastric mucosa visualized in the setting of retained gastric food contents could have provided information on the quality and adequacy of an examination, but this was not possible, given the retrospective study design. Finally, respiratory adverse events may have been detected more readily in the inpatient setting while some self-limited minor adverse events in the outpatient setting may have been missed, which could potentially explain the high association between respiratory adverse events and inpatient status. To circumvent this limitation, respiratory adverse events were clearly defined as the presence of acute symptoms with associated abnormal radiologic tests.

In conclusion, our study revealed that complete examination to the distal duodenum was achieved in nearly 90% of patients who were found to have unexpected gastric content encountered during endoscopy. However, substantial variation in completion rate was observed among individual endoscopists. The presence of large food burden decreased the likelihood of complete endoscopic examination after adjusting for endoscopist-specific completion rate. Furthermore, respiratory adverse events after endoscopy were frequent and led to substantial morbidity. Finally, respiratory adverse events occurred almost exclusively in hospitalized patients and was more likely in patients with large compared with small gastric food content.

## CONFLICTS OF INTEREST

**Guarantor of the article:** John J. Kim, MD, MS.

**Specific author contributions:** J.J.K. and E.L. contributed to the study conception and design. Data collection and analysis were performed by E.L., Y.G., and J.J.K. The first draft of the manuscript was written by E.L., and all authors commented on previous versions of the manuscript. All authors read and approved the final manuscript.

**Financial support:** None to report.

**Potential competing interests:** None to report.

**IRB approval statement:** This study was approved by the Institutional Review Board of Loma Linda University Health (IRB #5190288).

**Study Highlights**

**WHAT IS KNOWN**

✓ Retained gastric food contents are frequently encountered during upper endoscopy.
✓ The effects of retained gastric food content on clinical outcomes and endoscopic practice patterns are poorly understood.

**WHAT IS NEW HERE**

✓ Most patients received a complete examination despite gastric food contents.
✓ Rates of complete examination varied substantially by individual endoscopist and by the extent of food burden.
✓ Respiratory adverse events after endoscopy occurred almost exclusively in hospitalized patients and were associated with a high morbidity.
